# Prevalence of Vitamin D Deficiency Varies Widely by Season in Canadian Children and Adolescents with Sickle Cell Disease

**DOI:** 10.3390/jcm7020014

**Published:** 2018-01-30

**Authors:** Kaitlyn L. I. Samson, Heather McCartney, Suzanne M. Vercauteren, John K. Wu, Crystal D. Karakochuk

**Affiliations:** 1 Food, Nutrition, and Health, University of British Columbia, Vancouver, BC V6T 1Z4, Canada; kaitlyn.samson@ubc.ca; 2 British Columbia Children’s Hospital Research Institute, Vancouver, BC V6H 3N1, Canada; svercauteren2@cw.bc.ca (S.M.V.); jwu@bcchr.ca (J.K.W.); 3 Division of Hematology and Oncology, British Columbia Children’s Hospital, Vancouver, BC V6H 3N1, Canada; hmccartney@cw.bc.ca; 4 Division of Hematopathology, British Columbia Children’s Hospital, Vancouver, BC V6H 3N1, Canada; 5 Department of Pathology and Laboratory Medicine, University of British Columbia, Vancouver, BC V6T 1Z4, Canada; 6 Department of Paediatrics, University of British Columbia, Vancouver, BC V6T 1Z4, Canada

**Keywords:** sickle cell disease, vitamin D, 25-hydroxyvitamin D, pediatrics, nutrition, deficiency, hemoglobinopathy

## Abstract

Sickle cell disease (SCD) is an inherited disorder caused by a variant (*rs*334) in the β-globin gene encoding hemoglobin. Individuals with SCD are thought to be at risk of vitamin D deficiency. Our aim was to assess serum 25-hydroxyvitamin D (25OHD) concentrations, estimate deficiency prevalence, and investigate factors associated with 25OHD concentrations in children and adolescents with SCD attending BC Children’s Hospital in Vancouver, Canada. We conducted a retrospective chart review of SCD patients (2–19 y) from 2012 to 2017. Data were available for *n* = 45 patients with *n* = 142 25OHD measurements assessed using a EUROIMMUN analyzer (EUROIMMUN Medizinische Labordiagnostika AG, Lübeck, Germany). Additional data were recorded, including age, sex, and season of blood collection. Linear regression was used to measure associations between 25OHD concentration and predictor variables. Overall, mean ± SD 25OHD concentration was 79 ± 36 nmol/L; prevalence of low 25OHD concentrations (<30, <40, and <75 nmol/L) was 5%, 17% and 50%, respectively. Mean 25OHD concentrations measured during Jul–Sep were higher (28 (95% confidence interval CI: 16–40) nmol/L higher, *P* < 0.001) compared to Jan–Mar. Vitamin D deficiency rates varied widely by season: Based on 25OHD <30 nmol/L, prevalence was 0% in Oct–Dec and 6% in Jan–Mar; based on <40 nmol/L, prevalence was 0% in Oct–Dec and 26% in Jan–Mar.

## 1. Introduction

Sickle cell disease (SCD) is an inherited disorder [1,2] caused by a variant (*rs*334) in the β-globin gene encoding hemoglobin. It is one of the most common and severe monogenic disorders worldwide [2,3]. Mutation of the *rs*334 nucleotide from a thymine to an adenine base pair produces a hydrophobic motif, which, when deoxygenated, leads to polymerization and crystallization of the hemoglobin molecule, causing a sickle shape [2,3]. The severity of SCD is determined via the extent of polymerization, as sickled cells are rigid and inflexible. Sickling leads to vaso-occlusive crises, increased erythropoiesis and hemolysis, anemia, and further associated health complications [2,3]. Due to the increase in red cell turnover and basal metabolic rate (BMR), individuals with SCD are at increased risk of multiple nutrient deficiencies [4,5,6,7].

One nutrient of concern for individuals with SCD is vitamin D. Vitamin D plays an important role in cell growth and differentiation [8], cardiovascular health, immunity, and bone health [9,10]. It has been previously reported that patients with SCD have lower concentrations of 25-hydroxyvitamin D (25OHD) and an increased prevalence of vitamin D deficiency [7,11,12], which may be exacerbated by increased erythropoiesis and BMR [7], inadequate dietary intake [4,6], and decreased nutrient absorption due to inflammatory damage of the intestinal mucosa [13,14]. Additionally, the sickle cell variant is most commonly found in individuals of African-origin [15,16,17], thus, there is an increased risk of vitamin D deficiency in this population, as darker skin pigmentations absorb less ultraviolet B (UVB) radiation, reducing the skin’s production of vitamin D [9].

Due to this increased risk, the Canadian Haemoglobinopathy Association recommends daily supplementation of individuals with SCD with 1000–2000 IU vitamin D, following assessment of 25OHD concentrations [18]. However, a recent Cochrane review reported on the limited evidence of the effectiveness of vitamin D supplementation on outcomes among individuals with SCD (only one study was included with moderate to low quality of evidence), and concluded that more research is needed in this area before clinical recommendations could be made [1]. We aimed to measure serum 25OHD concentrations (as a biomarker of vitamin D status), estimate the prevalence of vitamin D deficiency, and investigate factors associated with 25OHD concentrations in children and adolescents with SCD attending the British Columbia Children’s Hospital in Vancouver, Canada.

## 2. Materials and Methods

### 2.1. Study Design and Participants

A retrospective chart review was conducted among SCD patients attending the sickle cell clinic at British Columbia Children’s Hospital in Vancouver, Canada over the past 5-year period (2012–2017). Data were collected for *n* = 45 patients aged 2–19 y. All children and adolescents with SCD were living in British Columbia between the 49th and the 54th parallel. All children and adolescents with SCD attending the sickle cell clinic between 2012 and 2017 were included in the study. Ethical approval was received through the University of British Columbia/Children’s and Women’s Health Centre of British Columbia Research Ethics Board (CW17-0175/H17-00655).

### 2.2. Data Collection

Data were gathered through the hospital’s electronic charting system, as well as through archived patient charts. Information including the patient’s date of birth, sex, ethnicity, sickle cell genotype, medication history, and supplement history was collected into a database. Sickle cell genotype was categorized by homozygous sickle cell anemia (β^S^β^S^), hemoglobin SC disease (β^S^β^C^), and hemoglobin S/β-thalassemia. Any current medications and supplements were also recorded along with their corresponding doses. Weight (kg) and height (cm) measurements were recorded. Month of blood collection was noted and categorized into four seasons (groups): January to March (Jan–Mar), April to June (Apr–Jun), July to September (Jul–Sep), and October to December (Oct–Dec).

Serum 25OHD concentration was measured using a EUROIMMUN analyzer with the corresponding 25OHD Vitamin D ELISA (EUROIMMUN Medizinische Labordiagnostika AG, Lübeck, Germany) at the British Columbia Children’s Hospital Clinical Biochemistry Lab (Vancouver, BC, Canada). Quality controls and three levels of calibrators provided by the manufacturer were run in each assay. The British Columbia Children’s Hospital participates in the Vitamin D External Quality Assessment Scheme (DEQAS), an external quality control program for 25OHD measurement and has a Certificate of Proficiency during the time in which the current analyses were completed. A complete blood count was performed using a Sysmex XN hematology analyzer (Sysmex Corporation, Kobe, Japan), including measurement of hemoglobin concentration (g/L), red cell distribution width (RDW; % of red blood cell), and mean corpuscular volume (MCV; fL). Serum was assessed for zinc (μmol/L), copper (μmol/L), and selenium concentrations (μmol/L) using a NexION 350 ICP-MS (Perkin Elmer, Waltham, MA, USA). Ferritin concentration (μg/L) and alkaline phosphatase (ALP) activity (U/L) were measured using a Vitros^®^ 5600 (Ortho Clinical Diagnostics, Raritan, NJ, USA).

### 2.3. Data Analysis

Body mass index (BMI)-for-age z-scores were calculated using an online anthropometric calculator, based on the World Health Organization Growth Reference Charts [19]. Vitamin D deficiency was defined as a serum 25OHD concentration <40 nmol/L [20], while insufficiency was defined as <75 nmol/L, as per the Canadian Paediatric Society guidelines [21].

For chemical and clinical biomarkers, concentrations were reported as mean ± SD or median (interquartile range, IQR) depending on the distribution (normal or skewed, respectively). Serum 25OHD concentrations are expressed as nmol/L (to obtain values in ng/mL: Divide nmol/L by 2.5).

A multivariable linear regression model was used to measure the association between mean serum 25OHD concentration (continuous outcome variable based on all available 25OHD measurements) and independent predictor variables which were selected based on a crude vs. adjusted change-in-estimate of ≥10%, controlling for repeated-measures of individuals. The primary predictor variable was age (continuous, years) given that our population was between 2 and 19 years and it was necessary to control for the wide variation in this variable in our population. Predictor variables that were known or suspected to be associated with vitamin D status that were available (recorded in patient charts) were assessed for inclusion in the model: age, sex, hemoglobin concentration, MCV, RDW, zinc, copper, selenium, ferritin, ALP, BMI-for-age z-score, sickle cell genotype, and whether children were receiving hydroxyurea or antibiotics for asplenia prophylaxis (penicillin or amoxicillin).

An analysis of variance (ANOVA) model was used to predict the marginal means (95% CI) of 25OHD concentrations by season (for all serum 25OHD measurements recorded in the past 5-year in all individuals), controlling for age and repeated-measures of individuals. Bonferroni-adjusted comparisons were used to detect statistical differences in 25OHD concentrations across seasons (*P* < 0.05). Stata/IC 15.0 (StataCorp, College Station, TX, USA) was used for statistical analyses.

## 3. Results

### 3.1. Characteristics of the Studied Population

Data were available for *n* = 45 children and adolescents with SCD. Of these, *n* = 42 had at least one 25OHD measure. Among all children, a total of *n* = 142 25OHD measurements were recorded in the 5-year period. The mean ± SD age of participants was 11.4 ± 5.3 y (Table 1). Overall, 47% of the studied population were male (*n* = 21/45). Self-reported ethnicity included African, Caribbean, Latino, or South Asian, and overall, 87% (*n* = 39/45) of individuals were of African-origin. Overall, 78% (*n* = 35/45) of individuals were diagnosed with homozygous sickle cell disease (β^S^β^S^) genotype (the remaining 22% of individuals had hemoglobin SC disease or hemoglobin S/β-thalassemia genotypes). A total of 62% (*n* = 28/45) of individuals were prescribed hydroxyurea (between 600 and 1000 mg/d). All individuals were recommended vitamin D supplements (between 500 and 1000 IU/d).

### 3.2. Biochemical and Clinical Markers

Overall, mean ± SD 25OHD concentration was 79.1 ± 35.9 nmol/L; prevalence of vitamin D deficiency (<40 nmol/L) and insufficiency (<75 nmol/L) was 17% and 50%, respectively (Table 2) [20]. Median (IQR) ALP activity was 139 (84, 185) U/L. Mean ± SD concentrations of copper and selenium were 20.4 ± 4.9 and 1.31 ± 0.17 μmol/L, respectively.

### 3.3. Factors Associated with Serum 25OHD Concentrations

Season, hemoglobin concentration, and ALP activity were significantly associated with serum 25OHD concentrations in children and adolescents (2–19 y) with SCD, after adjustment for confounding variables and repeated-measures of individuals (Table 3). Mean 25OHD concentrations assessed during the months of Jul–Sep were significantly higher (28 (95% CI: 16–40) nmol/L higher, *P* < 0.001), as compared to Jan–Mar. A 1 g/L increase in hemoglobin concentration was associated with a 0.4 (95% CI: 0.1, 0.8) nmol/L increase in mean serum 25OHD concentration (*P* = 0.01). A 1 U/L increase in ALP activity was associated with a 0.1 (95% CI: 0.1, 0.2) nmol/L increase in mean serum 25OHD concentration (*P* = 0.03).

### 3.4. Vitamin D Concentration by Season of Blood Collection

A total of 35 individuals had ≥2 serum measurements of 25OHD concentration in the 5-year period. Of those 35 individuals, *n* = 27 (77%) had a difference of ≥20 nmol/L between two of the measured values. The mean difference between the lowest and highest 25OHD measurements in all 35 individuals was 35.5 ± 20.2 nmol/L; overall, the individual differences ranged between 2 and 90 nmol/L (data not shown).

Mean serum 25OHD concentrations varied by season of blood collection, as did the prevalence of vitamin D deficiency and insufficiency. Prevalence of 25OHD <40 nmol/L varied by up to 26%, depending on the season of blood collection (26% in Jan–Mar and 0% in Oct–Dec). Similarly, the prevalence of vitamin D insufficiency (<75 nmol/L) varied by up to 36%, depending on the season of blood collection (38% in Jul–Sep vs. 74% in Jan–Mar) (Table 4).

**Figure 1:**
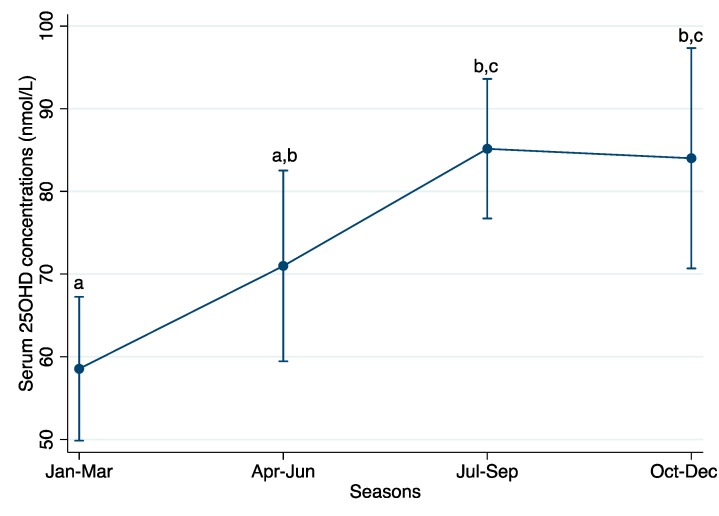
Marginal mean (95% CI) serum 25OHD concentrations by season based on *n* = 145 serum 25OHD measurements collected in the past 5-year period (2012–2017). Number of samples measured in each season were: *n* = 47 in Jan–Mar; *n* = 28 in Apr–Jun; *n* = 50 in Jul–Sep; and *n* = 20 in Oct–Dec. Values are marginal means (95% CI). An analysis of variance (ANOVA) model was used to predict the marginal means (95% CI) of 25OHD concentrations by season, controlling for age and repeated-measures of individuals. Values that do not share a common superscript letter in the column are significantly different from each other (Bonferroni-adjusted for multiple comparisons, *P* < 0.0125 to account for the four-group comparison).

Mean 25OHD concentrations collected in Jul–Sep and Oct–Dec were similar and both significantly higher as compared to in Jan–Mar, but not in Apr–Jun (Bonferroni-adjusted, *P* < 0.0125 to account for the four-group comparison) (Figure 1). Marginal mean (95% CI) serum 25OHD concentrations were 58.5 (49.8, 67.2) nmol/L in Jan–Mar, 71.0 (59.5, 82.5) nmol/L in Apr–Jun, 85.2 (76.7, 93.6) nmol/L in Jul–Sep, and 84.0 (70.7, 97.3) nmol/L in Oct–Dec.

## 4. Discussion

In this population of children and adolescents with SCD, who were predominately of African-origin, living in British Columbia, Canada, and were recommended daily vitamin D supplements (500–1000 IU/d), the prevalence of low serum 25OHD concentrations (<30, <40, and <75 nmol/L) was 5%, 17% and 50%, respectively, based on the individual’s most recent measure of serum 25OHD. Serum 25OHD concentrations measured in the summer months of Jul–Sep were significantly higher (28 (95% CI: 16–40) nmol/L higher, *P* < 0.001) than those collected in the winter months of Jan–Mar, highlighting the wide variation in mean 25OHD concentration by season.

The mean serum 25OHD concentrations in our studied population of SCD children and adolescents were higher than those observed in a nationally representative sample of healthy Canadian children (as per the Canadian Health Measures Survey 2007–2009) [22]. Albeit, comparisons among these two population groups are not justified given the major differences among children (e.g., disease-state of SCD, vitamin D supplementation practices, etc). Comparatively, healthy Canadian children aged 6–11 years had mean serum 25OHD concentrations of 75.0 (95% CI: 70.3–79.9) nmol/L and those aged 12–19 years had a mean 25OHD of 68.1 (95% CI: 63.8–72.4) nmol/L. Of note, we reiterate that all individuals with SCD in our study were recommended daily vitamin D supplements and we speculate that supplementation was likely one reason for the relatively high 25OHD concentrations observed in this population.

It is well-established that winter season is associated with lower serum 25OHD concentrations [23]. At latitudes of 35° N and above, the zenith angle at which the UVB photons hit the ozone layer during the winter months (November to February) causes a reduced amount of UVB to pass through the ozone, thus leading to reduced vitamin D synthesis in the skin [24]. Further, in dark-skinned individuals, high levels of epidermal melanin compete with 7-dehydocholesterol for UVB photons, decreasing the efficiency of vitamin D synthesis [9,24]. Thus, in our study, we were surprised to find seasonal changes in 25OHD concentrations in children of African-origin living at latitudes between 49 and 54° N.

Similar to our study, George et al. also observed that 25OHD deficiency prevalence varied by season (40% in winter, 31% in spring, 30% in summer, and 4% in autumn) in a healthy population of African adults residing in Johannesburg, South Africa (latitude: 26° S) [25], suggesting that despite the reduced vitamin D synthesis in the skin in individuals of African-origin, potential still exists for variation in 25OHD concentrations by season. Buison et al. also observed an association between season and 25OHD concentrations in children with SCD in the USA [26]. A multicenter cross-sectional survey conducted in England and the USA found that seasonal variation in 25OHD concentrations was observed in a pediatric SCD population (based on median 25OHD concentrations), but season had no effect on the prevalence of deficiency [27]. Conversely, limited seasonal variation in adult populations with SCD has been observed. We note; however, variation in 25OHD concentrations likely depends on multiple factors such as the overall vitamin D status of the population, dietary intakes of vitamin D, the latitude at which a population resides, and sun exposure [28,29].

Another significant predictor of serum 25OHD concentration in our model was ALP activity. Typically, in vitamin D deficiency, serum ALP activity levels are elevated, as ALP is released from osteoclasts during the process of bone demineralization [30]. Despite this, we found a significant positive association between serum ALP and 25OHD concentration. As SCD affects multiple systems throughout the body, and because ALP is secreted from tissues other than bone, ALP activity could be a result of other health-related complications (such as hepatic sequestration crisis and progressive cholestasis), rather than due to bone turnover and vitamin D status [18]. Given our limited available data in this retrospective chart review, we were unable to investigate this unexpected association further.

All children were recommended vitamin D supplements (500–1000 IU/d). However, we did not have data on adherence, as this information was not collected during regular patient visits. SCD is a chronic disorder often requiring several oral medications for its clinical management (e.g., hydroxyurea, penicillin, and folic acid). Further, vitamin D supplements are not often covered through health care plans. Therefore, the cost of the supplements and associated pill burden may negatively influence adherence rates among children and adolescents in our study. In summary, due to our lack of data on adherence, we could not assess the relationship between supplement use and serum 25OHD concentrations in our study.

More research is needed to investigate the effect of vitamin D supplementation on individuals with SCD, as they are a group that is particularly vulnerable to bone disorders and poor growth trajectories. To date, there has been only one randomized controlled trial on vitamin D supplementation in children with SCD (*n* = 42 were initially enrolled but only *n* = 37 completed the six month follow up to the trial) [31]. In the Osunkwo et al. trial, Vitamin D supplementation (40,000–100,000 IU/wk) caused a significant increase in 25OHD concentrations in the treatment group as compared to the placebo group; however, there was not sufficient evidence in improvement of clinical outcomes in children to guide clinical practice [31]. However, the dose of vitamin D provided in this supplementation trial was up to 10× the dose typically prescribed to children with SCD [31]. Additional studies have found that vitamin D supplementation was associated with increased 25OHD concentrations in SCD patients [32], as well as pain resolution [33], and improved bone mineral density [33,34]. However, a test of the safety and efficacy of high dose vitamin D supplementation (4000 IU vs. 7000 IU/d) in children and young adults found that neither dose was high enough to achieve the defined efficacy criterion (>32 ng/ml (equivalent to ~80 nmol/L)) in 80% of subjects after 12 weeks [35]. However, in individuals with the homozygous β^S^ sickle cell disease genotype, significant (*P* < 0.05) increases in fetal hemoglobin, decreases in high-sensitivity C-reactive protein, and decreases in platelet count were observed [35]. In conclusion, more high-quality controlled trials are needed on vitamin D supplementation in this population to guide clinical practice.

Some limitations should be considered when interpreting our results. First, only two markers of vitamin D status (serum 25OHD concentrations and ALP activity) were measured and recorded in patient charts. Additional biomarkers of vitamin D status, such as parathyroid hormone and vitamin D-binding protein, would have been useful for a more comprehensive assessment of vitamin D status [36]. Moreover, information on bone mineral density and inflammatory markers would also aid in the assessment of vitamin D status. Severe vitamin D deficiency is associated with lower bone mineral density, and vitamin D-binding protein is mildly affected by inflammation (e.g., 25OHD concentrations may decrease in the presence of inflammation) [7,9]. In addition, our studied population included only 42 individuals with 25OHD measurements; thus, we may have had limited power to detect significant associations in our linear regression model. Dietary intake data of children and adolescents were not collected during clinic visits; thus, we could not estimate dietary intakes of vitamin D in this population. Future research in this population group could include components of dietary intake assessment for a more comprehensive approach to assessing vitamin D status. A total of seven children were reported to have had a prior blood transfusion, which would influence serum 25OHD measurement if the sample was taken in approximately the ~3 weeks prior to the transfusion. Furthermore, a comparative group of age, sex, and ethnicity-matched controls would be useful to more rigorously compare 25OHD concentrations among children and adolescents with and without SCD living in the same geographical area.

In conclusion, the findings of this study highlight the importance of the season of blood collection when interpreting 25OHD concentrations, even among dark-skinned individuals with SCD living in northern latitudes. This information is important for clinicians when interpreting 25OHD concentrations in different seasons, as an individual classified as deficient in one month may not be deficient year-round (or vice versa).

## Figures and Tables

**Table 1:** Characteristics of Canadian children and adolescents (2–19 y) with sickle cell disease ^1^.

Characteristics	*n* (%)
**Sex**	
Males	21/45 (47%)
Females	24/45 (53%)
**Ethnicity**	
African	39/45 (87%)
Caribbean	3/45 (7%)
South Asian	2/45 (4%)
Latino	1/45 (2%)
**Sickle cell genotypes**	
Homozygous sickle cell anemia (β^S^β^S^)	35/45 (78%)
Hemoglobin S/β-thalassemia	7/45 (15%)
Hemoglobin SC disease (β^S^β^C^)	3/45 (7%)
**Currently receiving disease-modifying treatments**	
Blood transfusions	7/45 (16%)
Hydroxyurea (dose ranged from 400 to 1500 mg/d)	28/45 (62%)
Prophylaxis antibiotics (penicillin or amoxicillin)	37/45 (82%)
**Currently recommended nutritional supplements**	
Vitamin D (dose ranged from 500 to 1000 IU/d)	45/45 (100%)
Folic acid (dose ranged from 1 to 5 mg/d)	44/45 (98%)

Notes: ^1^ Total *n* = 45 children and adolescents; mean ± SD age was 11.4 ± 5.3 years.

**Table 2:** Biochemical and clinical markers among Canadian children and adolescents (2–19 y) with sickle cell disease ^1^.

Markers	All	Male	Female
**Total children and adolescents, *n* (%)**	45 (100%)	21 (47%)	24 (53%)
**Anthropometric indicators**			
Weight, kg	40.4 ± 18.4	37.9 ± 21.3	42.4 ± 16.0
Height, cm	140.6 ± 23.3	134.7 ± 28.1	145.2 ± 18.0
BMI-for-age, z-score, *n* (%)			
≥+2	3/42 (7%)	2/19 (11%)	1/23 (4%)
+1 to +2	10/42 (24%)	4/19 (21%)	6/23 (26%)
−1 to +1	26/42 (62%)	12/19 (63%)	14/23 (61%)
<−1	3/42 (7%)	1/19 (5%)	2/23 (9%)
**Nutritional indicators**			
25OHD concentration, nmol/L	79.1 ± 35.9	77.2 ± 38.1	80.7 ± 34.7
Prevalence of low 25OHD, *n* (%)			
<30 nmol/L	2/42 (5%)	1/19 (5%)	1/23 (4%)
<40 nmol/L	7/42 (17%)	3/19 (16%)	4/23 (17%)
<50 nmol/L	10/42 (24%)	5/19 (26%)	5/23 (22%)
<75 nmol/L	21/42 (50%)	10/19 (53%)	11/23 (48%)
ALP activity, U/L, median (IQR)	139 (84, 185)	149 (90, 185)	130.5 (76.5, 186)
Copper, μmol/L	20.4 ± 4.9	20.5 ± 5.0	20.3 ± 4.9
Selenium, μmol/L	1.31 ± 0.17	1.28 ± 0.18	1.34 ± 0.17
Zinc, μmol/L	11.3 ± 1.8	12.1 ± 2.0	10.7 ± 1.5
**Hematological indicators**			
Hemoglobin concentration, g/L	92.3 ± 16.5	93.0 ± 17.4	91.8 ± 16.2
Anemia, <110 g/L, *n* (%)	35/43 (81%)	14/19 (74%)	21/24 (88%)
Mean corpuscular volume, fL	85.0 ± 14.9	81.8 ± 11.7	87.6 ± 16.8
Red blood cell distribution width, %	19.3 ± 5.9	19.9 ± 6.2	18.8 ± 5.7
Ferritin, μg/L, median (IQR)	69 (45, 97)	67 (19, 95)	69 (46, 124)

Notes: ^1^ Total *n* = 45 children and adolescents (*n* = 42 had at least one measure of 25OHD). Values are mean ± SD unless otherwise indicated as *n* (%) or median (IQR). ALP, alkaline phosphatase; BMI, body mass index; IQR, interquartile range.

**Table 3:** Factors associated with serum 25OHD concentrations in Canadian children and adolescents (2–19 y) with sickle cell disease ^1^.

Associated Factors	25OHD Concentrations (nmol/L)		
	Beta-Coefficient (95% CI)	Standardized Beta	*P*
Age, y	−0.003 (−0.05, 0.04)	−0.01	0.90
Season (Ref: Jan–Mar)			
Apr–Jun	10.8 (−3.7, 25.2)	0.14	0.14
Jul–Sep	27.9 (15.8, 40.1)	0.41	<0.001
Oct–Dec	24.2 (8.3, 40.1)	0.26	0.003
Hemoglobin concentration, g/L	0.4 (0.1, 0.8)	0.20	0.01
ALP activity, U/L	0.1 (0.1, 0.2)	0.17	0.03
Receiving prophylaxis antibiotics ^2^ (Ref: no)	11.5 (−1.5, 24.4)	0.14	0.08
Constant	−10.1 (−45.5, 25.2)	NA	0.57

Notes: ^1^ Total *n* = 139 25OHD measurements from 42 children and adolescents (*n* = 6 values missing for ALP at random). Values are unstandardized (95% CI) and standardized beta coefficients. A multivariable linear regression model was used to measure the association between mean serum 25OHD concentration (continuous outcome variable) and independent predictor variables which were selected based on a crude vs. adjusted change-in-estimate of ≥10%, controlling for the repeated- measures of individuals. Adjusted R^2^ = 21%. ALP, alkaline phosphatase; CI, confidence interval; NA, not applicable; Ref, reference; ^2^ 82% of children (*n* = 37/45) were receiving prophylaxis antibiotics (penicillin (600–1000 mg/d) or amoxicillin (250–500 mg/d)).

**Table 4:** Prevalence of vitamin D deficiency and insufficiency among Canadian children and adolescents (2–19 y) with sickle cell disease based on mean serum 25OHD concentrations by season ^1^.

Serum 25OHD Concentrations				
Season	Deficiency ^2^ <30 nmol/L	Deficiency <40 nmol/L	Deficiency <50 nmol/L	Insufficiency ^3^ <75 nmol/L
Jan–Mar	3/47 (6%)	12/47 (26%)	17/47 (36%)	35/47 (74%)
Apr–Jun	1/28 (4%)	4/28 (14%)	7/28 (25%)	19/28 (68%)
Jul–Sep	3/50 (6%)	6/50 (12%)	10/50 (20%)	19/50 (38%)
Oct–Dec	0/20 (0%)	0/20 (0%)	0/20 (0%)	9/20 (45%)

Notes: ^1^ Total *n* = 145 serum 25OHD measurements collected in the past 5-year period (2012–2017). Values are *n* (%). Categories of deficiency prevalence are not mutually exclusive; in other words, each category includes all individuals with a 25OHD concentration below the specified cut-off.; ^2^ As defined by the Institute of Medicine [20]; ^3^ As defined by the Canadian Paediatric Society [21].

## References

[1] Soe H.H., Abas A.B., Than N.N., Ni H., Singh J., Said A.R., Osunkwo I. (2017). Vitamin D supplementation for sickle cell disease (Review). Cochrane Database Syst. Rev..

[2] Rees D.C., Williams T.N., Gladwin M.T. (2010). Sickle-cell disease. Lancet.

[3] Bunn F.H. (1997). Pathogenesis and Treatment of Sickle Cell Disease. N. Engl. J. Med..

[4] Gray N.T., Bartlett J.M., Kolasa K.M., Marcuard S.P., Holbrook C.T., Horner R.D. (1992). Nutritional status and dietary intake of children with sickle cell anemia. Am. J. Pediatr. Hematol. Oncol..

[5] Barden E.M., Zemel B.S., Kawchak D.A., Goran M.L., Ohene-Frempong K., Stallings V.A. (2000). Total and Resting Energy Expenditure in Children With Sickle Cell Disease. J. Pediatr..

[6] Hyacinth H.I., Gee B.E., Hibbert J.M. (2010). The Role of Nutrition in Sickle Cell Disease. Nutr. Metab. Insights.

[7] Nolan V.G., Nottage K.A., Cole E.W., Hankins J.S., Gurney J.G. (2015). Prevalence of Vitamin D deficiency in sickle cell disease: A systematic review. PLoS ONE.

[8] Nagpal S., Na S., Rathnachalam R. (2005). Noncalcemic actions of vitamin D receptor ligands. Endocr. Rev..

[9] Holick M.F. (2007). Vitamin D Deficiency. N. Engl. J. Med..

[10] DeLuca H.F. (2004). Overview of general physiologic features and functions of vitamin D. Am. J. Clin. Nutr..

[11] Martyres D.J., Vijenthira A., Barrowman N., Harris-Janz S., Chretien C., Klaassen R.J. (2016). Nutrient Insufficiencies/Deficiencies in Children With Sickle Cell Disease and Its Association With Increased Disease Severity. Pediatr. Blood Cancer.

[12] Rovner A.J., Stallings V.A., Kawchak D.A., Schall J.I., Ohene-Frempong K., Zemel B.S. (2008). High Risk of Vitamin D Deficiency in Children with Sickle Cell Disease. J. Am. Diet. Assoc..

[13] Julka R.N., Aduli F., Lamps L.W., Olden K.W. (2008). Ischemic duodenal ulcer, an unusual presentation of sickle cell disease. J. Natl. Med. Assoc..

[14] Phebus C.K., Maciak B.J., Gloninger M.F., Paul H.S. (1988). Zinc status of children with sickle cell disease: Relationship to poor growth. Am. J. Hematol..

[15] Flint J., Harding R.M., Boyce A.J., Clegg J.B. (1998). The population genetics of the haemoglobinopathies. Baillieres Clin. Haematol..

[16] Allison A.C. (1954). Protection Afforded By Sickle-Cell Trait against Subtertian Malarial Infection. Br. Med. J..

[17] Modell B., Darlison M. (2008). Global epidemiology of haemoglobin disorders and derived service indicators. Bull. World Health Organ..

[18] The Canadian Haemoglobinopathy Association (2015). The Canadian Haemoglobinopathy Association Consensus Statement on the Care of Patients with Sickle Cell Disease in Canada.

[19] Canadian Pediatric Endocrine Group (CPEG) WHO Growth Standard Charts. http://www.bcchildrens.ca/health-professionals/clinical-resources/endocrinology-diabetes/tools-calculators.

[20] Institute of Medicine (IOM) (2011). Dietary Reference Intakes for Calcium and Vitamin D.

[21] Godel J.C. (2007). Vitamin D supplementation: Recommendations for Canadian mothers and infants. Paediatr. Child Health.

[22] Langlois K., Greene-Finestone L., Little J., Hidiroglou N., Whiting S. (2010). Vitamin D status of Canadians as measured in the 2007 to 2009 Canadian Health Measures Survey. Health Rep..

[23] March K.M., Chen N.N., Karakochuk C.D., Shand A.W., Innis S.M., Von Dadelszen P., Barr S.I., Lyon M.R., Whiting S.J., Weiler H.A. (2015). Maternal vitamin D3 supplementation at 50 mg/d protects against low serum 25-hydroxyvitamin D in infants at 8 wk of age: A randomized controlled trial of 3 doses of vitamin D beginning in gestation and continued in lactation. Am. J. Clin. Nutr..

[24] Holick M.F. (2004). Vitamin D: Importance in the prevention of cancers, type 1 diabetes, heart disease, and osteoporosis. Am. J. Clin. Nutr..

[25] George J.A., Norris S.A., van Deventer H.E., Pettifor J.M., Crowther N.J. (2014). Effect of adiposity, season, diet and calcium or vitamin D supplementation on the vitamin D status of healthy urban African and Asian-Indian adults. Br. J. Nutr..

[26] Buison A.M., Kawchak D.A., Schall J., Ohene-Frempong K., Stallings V.A., Zemel B.S. (2004). Low vitamin D status in children with sickle cell disease. J. Pediatr..

[27] Jackson T.C., Krauss M.J., Debaun M.R., Strunk R.C., Arbeláez A.M. (2012). Vitamin D deficiency and comorbidities in children with sickle cell anemia. Pediatr. Hematol..

[28] Arlet J.B., Courbebaisse M., Chatellier G., Eladari D., Souberbielle J.C., Friedlander G., de Montalembert M., Prié D., Pouchot J., Ribeil J.A. (2013). Relationship between vitamin D deficiency and bone fragility in sickle cell disease: A cohort study of 56 adults. Bone.

[29] Goodman B.M., Artz N., Radford B., Chen I.A. (2010). Prevalence of vitamin D deficiency in adults with sickle cell disease. J. Natl. Med. Assoc..

[30] Elder C.J., Bishop N.J. (2014). Rickets. Lancet.

[31] Osunkwo I., Ziegler T.R., Alvarez J., McCracken C., Cherry K., Osunkwo C.E., Ofori-Acquah S.F., Ghosh S., Ogunbobode A., Rhodes J. (2012). High dose vitamin D therapy for chronic pain in children and adolescents with sickle cell disease: Results of a randomized double blind pilot study. Br. J. Haematol..

[32] Wykes C., Arasaretnam A., O’Driscoll S., Farnham L., Moniz C., Rees D.C. (2014). Vitamin D deficiency and its correction in children with sickle cell anaemia. Ann. Hematol..

[33] Osunkwo I. (2011). Complete resolution of sickle cell chronic pain with high dose vitamin D therapy: A case report and review of the literature. J. Pediatr. Hematol. Oncol..

[34] Adewoye A.H., Chen T.C., Ma Q., McMahon L., Mathieu J., Malabanan A., Steinberg M.H., Holick M.F. (2008). Sickle cell bone disease: Response to vitamin D and calcium. Am. J. Hematol..

[35] Dougherty K.A., Bertolaso C., Schall J.I., Smith-Whitley K., Stallings V.A. (2015). Safety and Efficacy of High-dose Daily Vitamin D3 Supplementation in Children and Young Adults With Sickle Cell Disease. J. Pediatr. Hematol. Oncol..

[36] Powe C.E., Evans M.K., Wenger J., Zonderman A.B., Berg A.H., Nalls M., Tamez H., Zhang D., Bhan I., Karumanchi S.A. (2013). Vitamin D-binding protein and vitamin D status of black Americans and white Americans. N. Engl. J. Med..

